# Lack of difference in thyroid hormone profile between offspring conceived naturally and through ART

**DOI:** 10.1093/hropen/hoag009

**Published:** 2026-02-09

**Authors:** Yiyuan Zhang, Yifei Sun, Ting Lan, Huang Wei, Wenjing Wan, Linlin Cui, Wei Zhou, Zi-Jiang Chen

**Affiliations:** Center for Reproductive Medicine, The Second Qilu Hospital of Shandong University, Jinan, Shandong, 250012, China; State Key Laboratory of Reproductive Medicine and Offspring Health, Center for Reproductive Medicine, Institute of Women, Children and Reproductive Health, Shandong University, Jinan, Shandong, 250012, China; National Research Center for Assisted Reproductive Technology and Reproductive Genetics, Shandong University, Jinan, Shandong, 250012, China; Key Laboratory of Reproductive Endocrinology (Shandong University), Ministry of Education, Jinan, Shandong, 250012, China; Shandong Technology Innovation Center for Reproductive Health, Jinan, Shandong, 250012, China; Shandong Provincial Clinical Research Center for Reproductive Health, Jinan, Shandong, 250012, China; Shandong Key Laboratory of Reproductive Research and Birth Defect Prevention, Jinan, Shandong, 250012, China; Research Unit of Gametogenesis and Health of ART-Offspring, Chinese Academy of Medical Sciences (No.2021RU001), Jinan, Shandong, 250012, China; Center for Reproductive Medicine, The Second Qilu Hospital of Shandong University, Jinan, Shandong, 250012, China; State Key Laboratory of Reproductive Medicine and Offspring Health, Center for Reproductive Medicine, Institute of Women, Children and Reproductive Health, Shandong University, Jinan, Shandong, 250012, China; National Research Center for Assisted Reproductive Technology and Reproductive Genetics, Shandong University, Jinan, Shandong, 250012, China; Key Laboratory of Reproductive Endocrinology (Shandong University), Ministry of Education, Jinan, Shandong, 250012, China; Shandong Technology Innovation Center for Reproductive Health, Jinan, Shandong, 250012, China; Shandong Provincial Clinical Research Center for Reproductive Health, Jinan, Shandong, 250012, China; Shandong Key Laboratory of Reproductive Research and Birth Defect Prevention, Jinan, Shandong, 250012, China; Research Unit of Gametogenesis and Health of ART-Offspring, Chinese Academy of Medical Sciences (No.2021RU001), Jinan, Shandong, 250012, China; Center for Reproductive Medicine, The Second Qilu Hospital of Shandong University, Jinan, Shandong, 250012, China; State Key Laboratory of Reproductive Medicine and Offspring Health, Center for Reproductive Medicine, Institute of Women, Children and Reproductive Health, Shandong University, Jinan, Shandong, 250012, China; National Research Center for Assisted Reproductive Technology and Reproductive Genetics, Shandong University, Jinan, Shandong, 250012, China; Key Laboratory of Reproductive Endocrinology (Shandong University), Ministry of Education, Jinan, Shandong, 250012, China; Shandong Technology Innovation Center for Reproductive Health, Jinan, Shandong, 250012, China; Shandong Provincial Clinical Research Center for Reproductive Health, Jinan, Shandong, 250012, China; Shandong Key Laboratory of Reproductive Research and Birth Defect Prevention, Jinan, Shandong, 250012, China; Research Unit of Gametogenesis and Health of ART-Offspring, Chinese Academy of Medical Sciences (No.2021RU001), Jinan, Shandong, 250012, China; Center for Reproductive Medicine, The Second Qilu Hospital of Shandong University, Jinan, Shandong, 250012, China; State Key Laboratory of Reproductive Medicine and Offspring Health, Center for Reproductive Medicine, Institute of Women, Children and Reproductive Health, Shandong University, Jinan, Shandong, 250012, China; National Research Center for Assisted Reproductive Technology and Reproductive Genetics, Shandong University, Jinan, Shandong, 250012, China; Key Laboratory of Reproductive Endocrinology (Shandong University), Ministry of Education, Jinan, Shandong, 250012, China; Shandong Technology Innovation Center for Reproductive Health, Jinan, Shandong, 250012, China; Shandong Provincial Clinical Research Center for Reproductive Health, Jinan, Shandong, 250012, China; Shandong Key Laboratory of Reproductive Research and Birth Defect Prevention, Jinan, Shandong, 250012, China; Research Unit of Gametogenesis and Health of ART-Offspring, Chinese Academy of Medical Sciences (No.2021RU001), Jinan, Shandong, 250012, China; Center for Reproductive Medicine, The Second Qilu Hospital of Shandong University, Jinan, Shandong, 250012, China; State Key Laboratory of Reproductive Medicine and Offspring Health, Center for Reproductive Medicine, Institute of Women, Children and Reproductive Health, Shandong University, Jinan, Shandong, 250012, China; National Research Center for Assisted Reproductive Technology and Reproductive Genetics, Shandong University, Jinan, Shandong, 250012, China; Key Laboratory of Reproductive Endocrinology (Shandong University), Ministry of Education, Jinan, Shandong, 250012, China; Shandong Technology Innovation Center for Reproductive Health, Jinan, Shandong, 250012, China; Shandong Provincial Clinical Research Center for Reproductive Health, Jinan, Shandong, 250012, China; Shandong Key Laboratory of Reproductive Research and Birth Defect Prevention, Jinan, Shandong, 250012, China; Research Unit of Gametogenesis and Health of ART-Offspring, Chinese Academy of Medical Sciences (No.2021RU001), Jinan, Shandong, 250012, China; Center for Reproductive Medicine, The Second Qilu Hospital of Shandong University, Jinan, Shandong, 250012, China; State Key Laboratory of Reproductive Medicine and Offspring Health, Center for Reproductive Medicine, Institute of Women, Children and Reproductive Health, Shandong University, Jinan, Shandong, 250012, China; National Research Center for Assisted Reproductive Technology and Reproductive Genetics, Shandong University, Jinan, Shandong, 250012, China; Key Laboratory of Reproductive Endocrinology (Shandong University), Ministry of Education, Jinan, Shandong, 250012, China; Shandong Technology Innovation Center for Reproductive Health, Jinan, Shandong, 250012, China; Shandong Provincial Clinical Research Center for Reproductive Health, Jinan, Shandong, 250012, China; Shandong Key Laboratory of Reproductive Research and Birth Defect Prevention, Jinan, Shandong, 250012, China; Research Unit of Gametogenesis and Health of ART-Offspring, Chinese Academy of Medical Sciences (No.2021RU001), Jinan, Shandong, 250012, China; State Key Laboratory of Reproductive Medicine and Offspring Health, Center for Reproductive Medicine, Institute of Women, Children and Reproductive Health, Shandong University, Jinan, Shandong, 250012, China; National Research Center for Assisted Reproductive Technology and Reproductive Genetics, Shandong University, Jinan, Shandong, 250012, China; Key Laboratory of Reproductive Endocrinology (Shandong University), Ministry of Education, Jinan, Shandong, 250012, China; Shandong Technology Innovation Center for Reproductive Health, Jinan, Shandong, 250012, China; Shandong Provincial Clinical Research Center for Reproductive Health, Jinan, Shandong, 250012, China; Shandong Key Laboratory of Reproductive Research and Birth Defect Prevention, Jinan, Shandong, 250012, China; Research Unit of Gametogenesis and Health of ART-Offspring, Chinese Academy of Medical Sciences (No.2021RU001), Jinan, Shandong, 250012, China; Shanghai Key Laboratory for Assisted Reproduction and Reproductive Genetics, Shanghai, China; Department of Reproductive Medicine, Ren Ji Hospital, Shanghai Jiao Tong University School of Medicine, Shanghai, China

**Keywords:** ART, fresh embryo transfer, frozen embryo transfer, children, long-term health, thyroid function, thyroid stimulating hormone, free triiodothyronine, free thyroxine

## Abstract

**STUDY QUESTION:**

Does the thyroid function of children conceived through ART differ from that of naturally conceived (NC) children?

**SUMMARY ANSWER:**

Our study results indicate that both children conceived through fresh embryo transfer and frozen embryo transfer exhibit a thyroid function profile similar to that of NC children.

**WHAT IS KNOWN ALREADY:**

Few studies have compared thyroid function between ART-conceived and NC children, and conflicting conclusions have been reported.

**STUDY DESIGN, SIZE, DURATION:**

This study was based on a cohort at a tertiary health centre in China. The prospective cohort study included 9450 children born between 2005 and 2021 and followed up until June 2023. The outcomes of interest were the levels of the children’s thyroid-stimulating hormone (TSH), free triiodothyronine (FT3), free thyroxine (FT4), thyroglobulin autoantibody (A-TG), and thyroid peroxidase antibody (A-TPO).

**PARTICIPANTS/MATERIALS, SETTING, METHODS:**

The participants were 3940 fresh embryo transfer conceived children (ET group), 4730 frozen embryo transfer conceived children (FET group), and 780 NC children. Their ages ranged from 1.5 to 10 years. Generalized estimating equations (GEE) was used to compare the main outcomes.

**MAIN RESULTS AND THE ROLE OF CHANCE:**

No significant between-group differences were observed in FT4 levels or the odds of TSH, FT3, FT4, A-TG, or A-TPO levels beyond the standard range. Further subgroup analysis indicated that the ET group exhibited a slightly higher TSH level than the NC group during the toddler age period (mean TSH: 2.93 vs. 2.72 µIU/ml). However, this difference was no longer significant among preschool and school-aged children. Additionally, there were no significant differences in FT4 levels between the ART and NC groups across all age subgroups.

**LIMITATIONS, REASONS FOR CAUTION:**

Due to the inability to acquire urinary iodine data, we cannot adequately assess the influence of iodine intake on the study results. Both the unavoidable loss to follow-up in the cohort and the imbalance in the proportion of girls (higher in the NC group than in the ART group) may potentially introduce selection bias.

**WIDER IMPLICATIONS OF THE FINDINGS:**

The study results indicate that ART-conceived children exhibit a thyroid function profile comparable to that of NC children. Furthermore, ART appears to be safe for long-term thyroid function in children.

**STUDY FUNDING/COMPETING INTEREST(S):**

National Key Research and Development Program of China (no. 2024YFC2706700), the National Natural Science Foundation of China (NSFC) Regional Innovation and Development Joint Fund (U24A20664), the National Key Technology Research and Developmental Program of China (2024YFC2706902), CAMS Innovation Fund for Medical Sciences (2021-I2M-5-001), National Special Support Programme for High-level Talents, Taishan Scholars Programme for Young Experts of Shandong Province (tsqn202507388), Shandong Provincial Postdoctoral Innovation Project (no. SDCX-ZG-202502002), General Programme of Shandong Provincial Natural Science Foundation of China (ZR2022MH087), Young Talent of Lifting engineering for Science and Technology in Shandong, China (SDAST2024QTA071), Chinese Red Cross Foundation (2220025007). The authors declare no competing interests.

**TRIAL REGISTRATION NUMBER:**

N/A.

WHAT DOES THIS MEAN FOR PATIENTS? The aims of the present study were to determine whether the thyroid function of children conceived via assisted reproductive technology (ART) differs from that of children conceived naturally. Few studies have compared thyroid function between ART-conceived and naturally conceived (NC) children, and conflicting conclusions have been reported. This observational study included 3940 fresh embryo transfer conceived children (ET group), 4730 frozen embryo transfer conceived children (FET group), and 780 NC children. They were born between 2005 and 2021 and were followed up until June 2023. We determined the serum concentrations of the children’s thyroid-stimulating hormone (TSH), free triiodothyronine (FT3), free thyroxine (FT4), anti-thyroglobulin autoantibody (A-TG), and thyroid peroxidase autoantibody (A-TPO): all indicators of thyroid function. The study results indicated that ART-conceived children exhibit a thyroid function profile comparable to that of NC children. Furthermore, ART appears to be safe for long-term thyroid function in children.

## Introduction

More than 8 million children worldwide are born through ART, and this number is expected to continue to rise (Fauser, 2019). Children conceived via ART may be exposed to parental infertility and its associated comorbidities, as well as ART-related procedures such as controlled ovarian hyperstimulation, embryo or gamete manipulation, and transplantation (Fraser, 1993; Hesla, 1993). Controlled ovarian hyperstimulation in ART, particularly during fresh embryo transfer cycles, can result in unphysiologically elevated levels of maternal oestrogen during early pregnancy (Järvelä *et al.*, 2014). Oestrogen’s ability to promote thyroid cell growth has been validated in both humans and animals (Furlanetto *et al.*, 1999; Manole *et al.*, 2001). Human thyroid gland morphogenesis commences as early as the third week of pregnancy (Nilsson and Fagman, 2017). Consequently, the thyroid function of offspring conceived through ART may be affected. Emerging evidence suggests that embryo freezing and ICSI may affect the long-term health of offspring by influencing epigenetic reprogramming (Chen *et al.*, 2020). Additionally, ART is associated with an increased risk of low birth weight (LBW) and preterm birth (PTB) (Pinborg *et al.*, 2013; Berntsen *et al.*, 2019; Elias *et al.*, 2020). Studies have confirmed a significantly higher prevalence of hypothyroidism among premature infants and low birth weight new-borns than among their full-term counterparts (Franco *et al.*, 2013; Korkmaz *et al.*, 2018; Liu *et al.*, 2020; Zdraveska and Kocova, 2021). These findings have intensified concerns regarding the thyroid function of children conceived via ART.

Thyroid hormones are crucial and exert effects on almost all tissues. In childhood, thyroid and growth hormones coordinate to promote growth and development (Bassett and Williams, 2016) and to widely regulate energy metabolism and substance anabolism and catabolism (Song *et al.*, 2011). Few studies have compared thyroid function between children via ART and those conceived naturally, and conflicting conclusions have been reported (Sakka *et al.*, 2009; Onal *et al.*, 2012; Androulakis *et al.*, 2014; Ping-Ping Lv, 2014; Wijs *et al.*, 2022). Some research has indicated that children conceived through ART exhibit signs of hyperthyroidism, characterized by elevated levels of free triiodothyronine (FT3) and/or free thyroxine (FT4) (Androulakis *et al.*, 2014; Ping-Ping Lv, 2014; Wijs *et al.*, 2022), while other studies have noted that ART-conceived children have an increased risk of hyperthyroidism or subclinical hypothyroidism, characterized by elevated levels of thyroid-stimulating hormone (TSH) (Sakka *et al.*, 2009; Onal *et al.*, 2012; Ping-Ping Lv, 2014). Moreover, most studies have included children across a wide age range (e.g. 4–14 years) (Sakka *et al.*, 2009; Onal *et al.*, 2012; Ping-Ping Lv, 2014), preventing analysis by age group. It thus remains unclear when differences in thyroid function between ART-conceived and naturally conceived children manifest, and whether these changes decrease or increase with growth.

Therefore, the aims of the present study are to determine whether the thyroid function of children conceived via ART differs from that of children conceived naturally and to ascertain whether thyroid function in ART-conceived children normalizes with growth.

## Materials and methods

### Ethical approval

All parents provided signed informed consent, and the study protocol was approved by the ethics committee at the Institute of Women, Children and Reproductive Health, affiliated to Shandong University.

### Study design and setting

This study was based on the cohort from the Institute of Women, Children and Reproductive Health, which is affiliated with Shandong University. The aim of the cohort is to evaluate the long-term health outcomes (at least until early adulthood) of individuals conceived through ART. From this prospective cohort, we synchronously recruited children conceived via ART at our institute (ART group), as well as naturally conceived children (NC group) at our institute and other hospitals affiliated with Shandong University, who served as natural controls. The offspring included in this study were born between 2005 and 2021 and were followed up until June 2023.

Singleton children who participated in a thyroid function profile examination were deemed eligible for inclusion in the study. However, children with the following circumstances were excluded: (i) those conceived via preimplantation genetic diagnosis, *in vitro* maturation, or gamete intrauterine transfer; (ii) those with congenital anomalies; (iii) those with autoimmune diseases, genetic chromosomal disorders, neurological disorders, diabetes, growth hormone deficiency, growth retardation, or tumours; (iv) those taking medication that affects thyroid function; (v) those older than 10 years; and (vi) those whose parents had thyroid disease, diabetes, or autoimmune diseases or a history of these diseases. Ultimately, 9450 children were included in our study (Supplementary Fig. S1).

### Exposure assessment

The primary exposure of interest was ART, which encompasses techniques such as controlled ovarian hyperstimulation, IVF, ICSI, embryo culture, vitrification, and embryo transfer. Naturally conceived children, which are defined as those whose parents had not undergone ART treatment, were regarded as natural controls. Data on fertilization methods, types of embryo transfer, and endometrial preparation protocol for frozen embryo transfer were obtained from medical records.

### Outcomes

Follow-up visits with the participating children were conducted during the toddler age (1.5–2.9 years), preschool age (3.0–5.9 years), and school age (6.0–10.0 years) (National People’s Congress, 2024). We advised the children to be followed up on at least once during each developmental stage. The outcomes of interest were the children’s TSH, FT3, and FT4 levels. These were included in the models as both continuous and binary variables. Anti-thyroglobulin antibody (A-TG) and anti-thyroid peroxidase antibody (A-TPO) were included in the models as binary variables. Fasting blood samples were collected by nurses in the morning and stored at –80°C until analysis. The TSH (µIU/ml), FT3 (pmol/l), FT4 (pmol/l), A-TG (U), A-TPO (U) levels were measured using automated chemiluminescence immunoassays (Cobas e601 instrument, Ichige, Hitachinaka-shi, Japan; Roche Diagnostics, Mannheim, Germany). The following reference ranges were established based on local population and laboratory data: TSH = 0.7–5.97 µIU/l, FT3 = 3.69–8.46 pmol/l, FT4 = 12.3–22.8 pmol/l for ages 1–6 years; TSH = 0.6–4.84 µIU/l, FT3 = 3.88–8.02 pmol/l, FT4 = 12.5–21.5 pmol/l for ages 7–10 years; A-TPO = 0–34U; A-TG = 0–115U. Hypertyrotropinemia was defined as a TSH level that exceeds the upper limit of the reference range.

### Covariates

Data on parental demographics, anthropometric data, medical history, and family socioeconomic status prior to pregnancy were collected. The parents’ medical history was defined by the presence of diagnoses such as diabetes, autoimmune diseases, and thyroid disorders. Family socioeconomic status was determined by the highest level of education attained by the parents. Education levels were categorized as 3 years of college or above, senior high school, and junior high school or below. Occupation was classified as student or unemployed, physical labour, or mental labour. Parity was categorized as first-born, second-born, or later-born. Furthermore, data on neonatal anthropometry, congenital malformations and diseases, hypertensive disorders of pregnancy (HDP), and gestational diabetes mellitus (GDM) were collected within 42 days following delivery. Congenital malformations in new-borns were determined according to the International Classification of Diseases, 10th Revision (ICD-10) (WHO, 2004). Tobacco exposure during pregnancy was defined as maternal smoking or smoking in the maternal living environment during pregnancy. Children’s demographics, anthropometric data, and medical history were recorded during each follow-up visit. Specifically, height (measured to the nearest 0.1 cm) and weight (measured to the nearest 0.1 kg) were obtained twice using a stadiometer and scale. All children were required to wear lightweight clothing during these measurements. The body mass index (BMI) of children was calculated as the weight divided by the height squared. The testicular volume (Tanner stages G1–G5) of boys and breast development of girls (Tanner stages B1–B5) were examined through physical examination by professional paediatricians, Tanner II–V were defined as puberty (Tanner, 1969, 1970). The children’s medical history was defined by the presence of diagnoses such as autoimmune diseases, genetic chromosomal disorders, nervous system diseases, diabetes, growth hormone deficiency, growth retardation, and tumours. The age and BMI of the children and the age of the parents were adjusted as continuous variables. Categorical covariates, including sex, parity, tobacco exposure during pregnancy, education level, and occupation, were converted into dummy variables prior to adjustment in the models.

### Statistical analysis

Normally distributed data are expressed as means ± standard deviations (SD), while skewed variables are presented as medians ± interquartile ranges. Categorical variables are expressed as numbers and percentages. The *t*-test and Wilcoxon rank-sum test were applied to continuous variables with normal and skewed distributions, respectively. The chi-square and Fisher’s exact tests were used to analyse categorical data.

At each follow-up stage, 7.8%–18.3% of the participants were visited more than once (Supplementary Tables S1 and S2). As the study was designed to collect repeated measurements, we used generalized estimating equations (GEE) to compare the main outcomes. Linear regression was applied to estimate the mean differences and 95% confidence intervals (CIs) for continuous variables. Logistic regression was used to estimate the odds ratios (ORs) and 95% CIs for binary variables. The covariates were determined based on previous studies (Korevaar *et al.*, 2016; Önsesveren *et al.*, 2017; Simon *et al.*, 2018; Luo *et al.*, 2020) or had been confirmed to affect both the exposure and outcome variables. We adjusted for the children’s age, sex, and BMI; parity; parental age; tobacco exposure during pregnancy; and socioeconomic factors. Sensitivity analysis was conducted using propensity score matching (PSM). Children in the ART and NC groups were evaluated using PSM methodology with nearest neighbour matching (calliper = 0.02). The matching ratio was set at 1:4, and the matching factors were the children’s age and sex and parental age (Supplementary Tables S3 and S4). We further conducted a sensitivity analysis involving children who participated in only one follow-up visit per age group (Supplementary Table S5). To ensure that our findings were not influenced by pubertal development, a sensitivity analysis was performed specifically on children in Tanner stage I (Supplementary Table S6). The required sample size per group was determined based on the mean and standard deviation of TSH levels between the ART and NC groups, as reported in existing literature. Using a two-tailed significance level (α  =  0.05) and statistical power (1 − β  =  0.80), we calculated the necessary sample size to detect clinically meaningful differences. The final estimated sample size requirement per group was 318 participants (Sakka *et al.*, 2009). We have included all eligible individuals in our cohort. The statistical power of the study was calculated to be 0.99, indicating that the sample size is sufficient to detect a meaningful effect.

All statistical analyses were performed using R version 4.0.3 (R Foundation for Statistical Computing, Vienna, Austria) and PASS version 21.0.3 (NCSS, LLC, Kaysville, UT, USA), with a two-tailed *α* of 0.05.

## Results

### Participant characteristics

This study included 3940 children in the fresh embryo transfer group (ET group), 4730 children in the frozen embryo transfer group (FET group), and 780 in the NC group (Supplementary Fig. S1). The birth, parental, and socioeconomic characteristics of the participants are shown in Table 1. Compared with the NC group, children in the ET and FET groups had older parents at the time of birth (ET vs. NC: mother’s mean age, 31.90 vs. 29.27 years; father’s mean age, 32.79 vs. 30.34 years; FET vs. NC: mother’s mean age, 31.68 vs. 29.27 years; father’s mean age, 32.40 vs. 30.34 years). Children in the ET and FET groups were also more frequently exposed to GDM (ET vs. NC: 6.9% vs. 2.4%; FET vs. NC: 8.1% vs. 2.4%) and HDP (ET vs. NC: 4.1% vs. 1.9%; FET vs. NC: 5.9% vs. 1.9%) and more likely to be the first child (ET vs. NC: 82.8% vs. 55.5%; FET vs. NC: 77.8% vs. 55.5%) than the NC group. Compared with children in the NC group, children in the ET and FET groups had a significantly lower mean gestational age (ET vs. NC: 39.04 vs. 39.33 weeks; FET vs. NC: 39.03 vs. 39.33 weeks), age (ET vs. NC: 4.29 vs. 5.16 years; FET vs. NC: 3.64 vs. 5.16 years), and proportion of girls (ET vs. NC: 48.4% vs. 60.6%; FET vs. NC: 47.1% vs. 60.6%) and the FET group had significantly higher birth weight (FET vs. NC: 3469.91 vs. 3412.16 g).

**Table 1.** Children’s characteristics, parental characteristics, and family socioeconomic status.

	NC (780)	ET (3940)	FET (4730)	ET vs NC	FET vs NC
				*P*-value	*P*-value
**Parental characteristics**					
Maternal age at delivery, y	29.27 ± 4.67	31.90 ± 4.47	31.68 ± 4.12	**<0.001**	**<0.001**
Paternal age at delivery, y	30.34 ± 5.10	32.79 ± 5.15	32.40 ± 4.66	**<0.001**	**<0.001**
GDM, n (%)	19 (2.4%)	270 (6.9%)	383 (8.1%)	**<0.001**	**<0.001**
HDP, n (%)	15 (1.9%)	160 (4.1%)	280 (5.9%)	**<0.001**	**<0.001**
Tobacco exposure during pregnancy	9 (1.2%)	124 (3.1%)	90 (1.9%)		
Parity, n (%)					
First born	433 (55.5%)	3264 (82.8%)	3682 (77.8%)	**<0.001**	**<0.001**
Second or later	337 (43.2%)	666 (16.9%)	1034 (21.9%)		
**Family socioeconomic status**					
Highest occupation					
Mental labour	255 (32.7%)	1266 (32.1%)	1562 (33.0%)	**0.015**	**<0.001**
Physical labour	509 (65.3%)	2592 (65.8%)	3033 (64.1%)		
Student or unemployed	13 (1.7%)	81 (2.1%)	135 (2.9%)		
Highest education					
College or above	385 (49.4%)	1761 (44.7%)	2094 (44.3%)	**0.027**	**0.013**
Senior high school	176 (22.6%)	1046 (26.5%)	1271 (26.9%)		
Junior high school or below	219 (28.1%)	1133 (28.8%)	1365 (28.9%)		
**Child characteristics**					
Girls, n (%)	473 (60.6%)	1,908 (48.4%)	2227 (47.1%)	**<0.001**	**<0.001**
Birth weight, g	3412.16 ± 463.06	3426.73 ± 513.87	3469.91 ± 515.51	0.462	0.003
Length, cm	50.35 ± 1.78	50.29 ± 1.99	50.27 ± 1.92	0.436	0.318
Gestational age, w	39.33 ± 1.36	39.04 ± 1.54	39.03 ± 1.56	**<0.001**	**<0.001**
Premature birth, n (%)	22 (2.8%)	235 (6.0%)	306 (6.5%)	**<0.001**	**<0.001**
Age, y	5.16 ± 2.69	4.29 ± 2.03	3.64 ± 1.73	**<0.001**	**<0.001**
1.5–2.9 y, n (%)	293 (30.0%)	1948 (32.5%)	3131 (45.5%)	**<0.001**	**<0.001**
3–5.9 y, n (%)	323 (33.1%)	2773 (46.2%)	2996 (43.5%)		
6–10 y, n (%)	360 (36.9%)	1281 (21.3%)	756 (11.0%)		
BMI, kg/m^2^	16.26 ± 2.11	16.26 ± 2.17	16.27 ± 2.10	0.967	0.852

Data presented as mean ± SD for continuous variables and n (%) for categorical variables.

ET, fresh embryo transfer; FET, Frozen embryo transfer; GDM: gestational diabetes mellitus; HDP, hypertensive disorders during pregnancy; NC, naturally conceived children.

Variables in bold indicate statistical significance (*P* ≤ 0.05).

### Thyroid function

Thyroid function data for the NC, ET, and FET groups are presented in Table 2. No significant between-group differences were observed in the FT4 levels nor in the odds of TSH, FT3, FT4, A-TG, and A-TPO levels exceeding the standard ranges (Table 2). However, after adjusting for all confounding factors, children in the ET group exhibited slightly higher TSH levels compared to the NC group (3.12 vs. 3.07 µIU/ml). In addition, children in the ET and FET groups had a slightly higher FT3 level than those in the NC group (ET vs. NC: 6.95 vs. 6.84 pmol/l; FET vs. NC: 7.01 vs. 6.84 pmol/l), even after adjusting for confounding variables.

**Table 2.** Differences in thyroid function between NC and ART children.

	NC	ET	FET	ET vs NC		FET vs NC	
				**Unadjusted** **β/OR 95%CI**	**Adjusted** **β/OR 95%CI**	**Unadjusted** **β/OR 95%CI**	**Unadjusted** **β/OR 95%CI**
n	976	6002	6883				
Age, y	5.16 ± 2.69	4.29 ± 2.03	3.64 ± 1.73				
TSH, µIU/ml	3.07 ± 1.40	3.12 ± 1.93	2.98 ± 1.41	0.07 (**−**0.04, 0.18)	**0.13 (0.01, 0.25)**	**−**0.07 (**−**0.17, 0.04)	0.03 (**−**0.07, 0.14)
FT3, pmol/l	6.84 ± 0.80	6.95 ± 0.86	7.01 ± 0.85	**0.11 (0.05, 0.17)**	**0.10 (0.04, 0.16)**	**0.16 (0.11, 0.22)**	**0.13 (0.07, 0.19)**
FT4, pmol/l	18.40 ± 2.14	18.57 ± 2.23	18.67 ± 2.09	0.15 (**−**0.01, 0.31)	0.09 (**−**0.08, 0.26)	**0.25 (0.09, 0.40)**	0.14 (**−**0.03, 0.31)
High TSH	62 (6.4%)	330 (5.5%)	272 (4.0%)	0.91 (0.65, 1.27)	1.21 (0.86, 1.70)	**0.65 (0.47, 0.91)**	1.00 (0.71, 1.41)
Low TSH	6 (0.6%)	27 (0.4%)	40 (0.6%)	0.73 (0.30, 1.77)	0.59 (0.21, 1.64)	0.94 (0.40, 2.22)	0.71 (0.25, 2.00)
High FT3	34 (3.5%)	251 (4.2%)	296 (4.3%)	1.15 (0.81, 1.64)	1.34 (0.92, 1.95)	1.19 (0.84, 1.69)	1.38 (0.95, 2.02)
Low FT3	0	5 (0.1%)	5 (0.1%)	**–**	**–**	–	–
High FT4	31 (3.2%)	188 (3.1%)	234 (3.4%)	0.99 (0.68, 1.45)	1.08 (0.73, 1.61)	1.07 (0.74, 1.56)	1.22 (0.82, 1.81)
Low FT4	1 (0.1%)	3 (0.05%)	2 (0.03%)	0.48 (0.05, 4.57)	1.00 (0.06, 15.58)	0.28 (0.02, 3.04)	0.85 (0.08, 8.85)
High A-TG	13 (1.3%)	44 (0.7%)	19 (0.3%)	0.55 (0.29, 1.06)	1.18 (0.54, 2.59)	**0.21 (0.10, 0.42)**	0.56 (0.25, 1.28)
High A-TPO	13 (1.3%)	103 (1.7%)	90 (1.3%)	1.34 (0.73, 2.46)	1.37 (0.71, 2.67)	1.03 (0.56, 1.89)	1.15 (0.60, 2.20)

A-TG, thyroglobulin autoantibody; A-TPO, thyroid peroxidase antibody; ET, fresh embryo transfer; FET, frozen embryo transfer; n, number of follow-up visits; TSH, thyroid stimulating hormone; FT3, free triiodothyronine; FT4, free tetraiodothyronine; Low, below the lower limit of the reference range; High, above the lower limit of the reference range.

Variables in bold indicate statistical significance (*P* ≤ 0.05); adjusted for children’s age and BMI, parity, parental age at delivery, socioeconomic factors, tobacco exposure during pregnancy.

To investigate age-related changes in the thyroid function profiles of offspring conceived through ART, we conducted an age subgroup analysis (Table 3). Among toddlers, the ET group exhibited a slightly significantly higher mean TSH level than the NC group (2.93 vs. 2.72 µIU/ml). However, the difference in TSH between the ET and NC groups was not significant among preschool and school-aged children. No significant differences in FT4 levels were observed between the ET vs NC groups or FET vs NC groups across any age subgroup. Among preschool-aged children, the ET and FET groups demonstrated a significantly higher mean FT3 level than the NC group (ET vs. NC: 7.00 vs. 6.82 pmol/l; FET vs. NC: 7.04 vs. 6.82 pmol/l). This between-group difference in FT3 levels was not significant among school-aged children. Importantly, no significant differences in TSH, FT3, and FT4 levels were observed between the ET vs NC groups or FET vs NC in school-aged children after matching the children’s age and sex and parents’ age. Additionally, sensitivity analyses restricted to children with single follow-up visits in per age subgroup produced similar results with those including all participants (Supplementary Table S5).

**Table 3.** Differences in thyroid function between NC and ART children stratified by whether the embryo is frozen.

				ET vs NC		FET vs NC	
	NC	ET	FET	Unadjusted β 95%CI	Adjusted β 95%CI	Unadjusted β 95%CI	Adjusted β 95%CI
**1.5–2.9 y, n**	293	1948	3131				
TSH, µIU/ml	2.72 ± 1.28	2.93 ± 1.41	2.80 ± 1.32	**0.21 (0.03, 0.40)**	**0.25 (0.07, 0.42)**	0.09 (**−**0.06, 0.24)	0.12 (**−**0.04, 0.28)
FT3, pmol/l	6.97 ± 0.86	7.00 ± 0.87	7.01 ± 0.81	0.03 (**−**0.08, 0.14)	0.02 (**−**0.09, 0.14)	0.05 (**−**0.06, 0.16)	0.04 (**−**0.07, 0.15)
FT4, pmol/l	18.51 ± 1.96	18.74 ± 2.53	18.68 ± 2.05	0.23 (**−**0.02, 0.49)	0.26 (**−**0.03, 0.54)	0.17 (**−**0.08, 0.41)	0.17 (**−**0.09, 0.43)
**3–5.9 y, n**	323	2773	2996				
TSH, µIU/ml	3.01 ± 1.28	3.17 ± 2.38	3.09 ± 1.47	0.17 (0, 0.34)	0.19 (**−**0.03, 0.41)	0.10 (**−**0.06, 0.25)	0.10 (**−**0.07, 0.28)
FT3, pmol/l	6.82 ± 0.78	7.00 ± 0.87	7.04 ± 0.89	**0.18 (0.09, 0.27)**	**0.19 (0.09, 0.29)**	**0.23 (0.13, 0.32)**	**0.22 (0.12, 0.32)**
FT4, pmol/l	18.60 ± 2.08	18.59 ± 2.08	18.73 ± 2.14	0 (**−**0.24, 0.24)	**−**0.03 (**−**0.30, 0.25)	0.14 (**−**0.10, 0.38)	0.12 (**−**0.16, 0.39)
**6–10 y, n**	360	1281	756				
TSH, µIU/ml	3.40 ± 1.52	3.28 ± 1.45	3.22 ± 1.48	**−**0.13 (**−**0.31, 0.06)	**−**0.09 (**−**0.31, 0.14)	**−**0.17 (**−**0.36, 0.03)	**−**0.18 (**−**0.41, 0.05)
FT3, pmol/l	6.76 ± 0.76	6.79 ± 0.79	6.86 ± 0.80	0.03 (**−**0.06, 0.12)	0.03 (**−**0.07, 0.14)	**0.10 (0, 0.20)**	0.06 (**−**0.05, 0.18)
FT4, pmol/l	18.14 ± 2.30	18.27 ± 2.02	18.41 ± 2.05	0.11 (**−**0.16, 0.38)	**−**0.06 (**−**0.35, 0.24)	0.10 (**−**0.20, 0.40)	0.05 (**−**0.26, 0.36)

Data presented as mean ± SD for continuous variables and n for categorical variables.

ET, fresh embryo transfer; FET, frozen embryo transfer; n, number of follow-up visits; TSH, thyroid-stimulating hormone.

Variables in bold indicate statistical significance (*P* ≤ 0.05); adjusted for children’s age and BMI, parity, parental age at delivery, socioeconomic factors, tobacco exposure during pregnancy.

A subgroup analysis by endometrial preparation protocol for frozen embryo transfer is presented in Table 4 and Supplementary Table S7. No significant differences in TSH and FT4 levels were observed between the FET natural cycle groups vs NC, or FET hormone replacement cycle groups vs NC groups across any age subgroup. A subgroup analysis by fertilisation method is presented in Table 5. No significant differences in TSH and FT4 levels were observed between children in the NC group and those conceived via IVF or ICSI groups across any age subgroup.

**Table 4.** Differences in thyroid function between NC and ART children.

	**NC**	FET-NC	FET-HRT	NC vs NC-FET		NC vs HRT-FET	
				**Unadjusted** **β 95%CI**	**Adjusted** **β 95%CI**	**Unadjusted** **β 95%CI**	**Adjusted** **β 95%CI**
**1.5 − 2.9 y, n**	293	1653	1122				
TSH, µIU/ml	2.72 ± 1.28	2.78 ± 1.31	2.80 ± 1.33	0.03 (**−**0.16, 0.22)	**−**0.07 (**−**0.27, 0.14)	0.05 (**−**0.15, 0.24)	**−**0.05 (**−**0.27, 0.15)
FT3, pmol/l	6.97 ± 0.86	7.01 ± 0.83	7.00 ± 0.81	0.02 (**−**0.10, 0.13)	0.05 (**−**0.05, 0.15)	0.01 (**−**0.11, 0.13)	0.03 (**−**0.07, 0.14)
FT4, pmol/l	18.51 ± 1.96	18.69 ± 2.11	18.69 ± 2.11	0.08 (**−**0.22, 0.37)	0.04 (**−**0.28, 0.35)	0.05 (**−**0.25, 0.34)	0 (**−**0.33, 0.33)
**3–5.9 y, n**	323	1545	1080				
TSH, µIU/ml	3.01 ± 1.28	3.07 ± 1.44	3.08 ± 1.50	0.07 (**−**0.12, 0.26)	0.02 (**−**0.19, 0.22)	0.08 (**−**0.11, 0.28)	**−**0.001 (**−**0.22, 0.22)
FT3, pmol/l	6.82 ± 0.78	7.00 ± 0.84	7.11 ± 0.99	**0.13 (0.02, 0.25)**	**0.14 (0.01, 0.27)**	**0.25 (0.13, 0.37)**	**0.22 (0.08, 0.35)**
FT4, pmol/l	18.60 ± 2.08	18.62 ± 2.06	18.85 ± 2.26	**−**0.01 (**−**0.26, 0.23)	0.005 (**−**0.26, 0.27)	0.22 (**−**0.04, 0.48)	0.26 (**−**0.02, 0.54)
**6–10 y, n**	360	342	249				
TSH, µIU/ml	3.40 ± 1.52	3.28 ± 1.52	3.22 ± 1.49	**−**0.13 (**−**0.36, 0.11)	**−**0.08 (**−**0.35, 0.20)	**−**0.23 (**−**0.49, 0.04)	**−**0.18 (**−**0.49, 0.12)
FT3, pmol/l	6.76 ± 0.76	6.89 ± 0.80	6.87 ± 0.78	**0.13 (0.01, 0.25)**	0.11 (**−**0.04, 0.27)	0.11 (**−**0.02, 0.24)	0.06 (**−**0.10, 0.21)
FT4, pmol/l	18.14 ± 2.30	18.52 ± 2.09	18.47 ± 2.03	**−**0.08 (**−**0.35, 0.20)	**−**0.05 (**−**0.46, 0.37)	**−**0.18 (**−**0.49, 0.12)	**−**0.05 (**−**0.46, 0.36)

Data presented as mean ± SD for continuous variables and n for categorical variables.

FT3, free triiodothyronine; FT4, free tetraiodothyronine; HRT-FET, hormone replacement therapy for frozen embryo transfer; n, number of follow-up visits; NC, naturally conceived children; NC-FET, natural cycle protocol for frozen embryo transfer; TSH, thyroid-stimulating hormone.

Variables in bold indicate statistical significance (*P* ≤ 0.05); Adjusted for children’s age and BMI, parity, parental age at delivery, socioeconomic factors, tobacco exposure during pregnancy.

**Table 5.** Differences in thyroid function between NC and ART children stratified by fertilization method.

				IVF vs NC		ICSI vs NC	
	NC	IVF	ICSI	**Unadjusted** **β 95%CI**	**Adjusted** **β 95%CI**	**Unadjusted** **β 95%CI**	**Adjusted** **β 95%CI**
**1.5–2.9 y, n**	293	3498	1581				
TSH, µIU/ml	2.72 ± 1.28	2.86 ± 1.37	2.83 ± 1.33	0.14 (**−**0.02, 0.31)	0.05 (**−**0.15, 0.25)	0.12 (**−**0.06, 0.29)	0.02 (**−**0.18, 0.23)
FT3, pmol/l	6.97 ± 0.86	7.00 ± 0.83	7.02 ± 0.85	0.03 (**−**0.08, 0.13)	0.01 (**−**0.11, 0.12)	0.05 (**−**0.06, 0.17)	0.04 (**−**0.09, 0.16)
FT4, pmol/l	18.51 ± 1.96	18.70 ± 2.10	18.70 ± 2.55	0.19 (**−**0.06, 0.43)	0.07 (**−**0.22, 0.37)	0.18 (**−**0.10, 0.45)	0.08 (**−**0.24, 0.41)
**3–5.9 y, n**	323	3990	1779				
TSH, µIU/ml	3.01 ± 1.28	3.13 ± 2.11	3.14 ± 1.56	0.13 (**−**0.03, 0.29)	0.08 (**−**0.14, 0.29)	0.14 (**−**0.02, 0.30)	0.10 (**−**0.10, 0.31)
FT3, pmol/l	6.82 ± 0.78	7.04 ± 0.87	6.99 ± 0.91	**0.22 (0.13, 0.30)**	**0.18 (0.07, 0.29)**	**0.17 (0.07, 0.27)**	**0.14 (0.03, 0.26)**
FT4, pmol/l	18.60 ± 2.08	18.69 ± 2.11	18.60 ± 2.11	0.10 (**−**0.14, 0.33)	0.08 (**−**0.21, 0.38)	0.01 (**−**0.23, 0.25)	**−**0.01 (**−**0.32, 0.29)
**6–10 y, n**	360	1370	667				
TSH, µIU/ml	3.40 ± 1.52	3.22 ± 1.42	3.33 ± 1.52	**−0.18 (−0.36, 0)**	**−**0.19 (**−**0.40, 0.03)	**−**0.08 (**−**0.28, 0.13)	**−**0.10 (**−**0.33, 0.13)
FT3, pmol/l	6.76 ± 0.76	6.82 ± 0.81	6.81 ± 0.77	0.06 (**−**0.03, 0.15)	0.02 (**−**0.09, 0.13)	0.05 (**−**0.05, 0.15)	0.01 (**−**0.10, 0.13)
FT4, pmol/l	18.14 ± 2.30	18.36 ± 2.05	18.23 ± 2.01	0.22 (−0.05, 0.48)	0.01 (−0.29, 0.31)	0.09 (−0.20, 0.37)	−0.10 (−0.42, 0.22)

Data presented as mean ± SD for continuous variables and n for categorical variables.

n, number of follow-up visits; NC, naturally conceived children; TSH, thyroid-stimulating hormone; FT3, free triiodothyronine; FT4, free tetraiodothyronine.

Variables in bold indicate statistical significance (*P* ≤ 0.05); Adjusted for children’s age and BMI, parity, parental age at delivery, socioeconomic factors, tobacco exposure during pregnancy.

## Discussion

In this prospective cohort study of children aged 1.5–10 years, we found no significant differences in FT4 levels and the risk of TSH and FT4 exceeding the standard ranges between children in the NC and ART groups. Although we observed a slightly higher TSH level among children in the ET group than among their NC counterparts during toddlerhood, this difference is expected to decrease with increasing age.

In clinical practice, the TSH level is a key indicator in hypothyroidism screening, while the FT4 level is used to detect hyperthyroidism; both metrics are highly sensitive. Our study did not identify any differences in the odds of TSH exceeding the standard ranges or FT4 levels in both children conceived from fresh embryo transfer and frozen embryo transfer aged 1.5–10 years. Further subgroup analyses revealed that although children in the ET group exhibited a slight increase in TSH levels during toddler age, this resolved as they aged. Consistent with our findings, Onal *et al.* observed elevated TSH levels in children aged 2–4 weeks (Onal *et al.*, 2012). Sophia *et al.* focused on children aged 4 to 14 years (ART N = 106) and found elevated TSH levels and an increased risk of hyperthyrotropinaemia in those conceived via ART (Sakka *et al.*, 2009). However, due to limitations in their sample size, they did not further investigate the age at which these changes in TSH levels occurred. In contrast, Wijs *et al.*, which focused on older children, found higher FT4 levels in ART children aged 14 or 20 years (ART N = 134) than in NC children (Wijs *et al.*, 2022). The authors of that study noted that the difference in FT4 levels between the IVF and NC groups was minimal, and although it reached statistical significance, its clinical significance was questionable. Lv *et al.* reported higher FT4 levels in umbilical cord blood from the ART group than in the NC group (ART N = 103) (Ping-Ping Lv, 2014). However, we did not find a statistically significant difference in FT4 levels between children conceived via ART (ET or FET) and those conceived naturally during toddlerhood. Stress during childbirth may lead to a transient increase in cord blood FT4 levels, and it remains unclear whether these levels normalize with age or merely exhibit a brief increase during the peripartum period.

Thyroid hormone disorders in childhood have been shown to affect growth and development, as well as nervous system development (Stathatos, 2012; Waung *et al.*, 2012; Williams and Bassett, 2018; Schneider Aguirre and Fuqua, 2019; Giannocco *et al.*, 2021). Prior studies have demonstrated that, despite thyroid function residing within the normal range, FT4 levels in the upper quartile are associated with increased risks of diabetes, hypertension, and all-cause mortality related to cardiovascular disease (Gu *et al.*, 2018; Birck *et al.*, 2022; Lang *et al.*, 2022). Previously, many clinicians had expressed concerns that, in addition to infertility and its comorbidities, non-physiological hormone stimulation and artificial manipulation during ART could influence thyroid function in offspring by altering the levels of oestrogen (Manole *et al.*, 2001; Banu *et al.*, 2002; Korevaar *et al.*, 2016), thyroid hormone (Mintziori *et al.*, 2011; Dhillon-Smith *et al.*, 2020; Luo *et al.*, 2020; Busnelli *et al.*, 2021; Zhang and Li, 2021; Concepción-Zavaleta *et al.*, 2023), and human chorionic gonadotropin (d’Hauterive *et al.*, 2022), as well as by altering the epigenetics of embryos and gametes (Tsai *et al.*, 2002; Mani *et al.*, 2020; Hernandez *et al.*, 2023). Our study results indicate that children conceived via ART experience only mild changes in thyroid function during toddler age, and these changes are expected to resolve with growth and development. Similar results observed in both the fertilization and embryo transfer method subgroup analyses further confirm the safety of ART with respect to children’s long-term thyroid function.

The primary strengths of this study include its status as the first study to facilitate a nuanced assessment of the effects of ART on children across various ages, supported by a large sample size and comprehensive subgroup analyses. Furthermore, this study has enabled distinct evaluations of the effects of different ART techniques on the thyroid function outcomes of ART-conceived children. The longitudinal design of the study is another strength, as it enabled an investigation of how differences in thyroid function between ART and NC children may evolve over time. However, our research has certain limitations. First, we were unable to obtain the participating children’s urinary iodine levels. Nevertheless, the Chinese government regulates the iodine content in table salt based on the nutritional iodine levels in various regions (PRC MoHot, 2011), and all participants in our study were permanent residents of Shandong province, which is expected to have an extremely low incidence of iodine deficiency. Furthermore, this factor is unrelated to the grouping of participants. Therefore, we speculate that the iodine level was unlikely to have a significant effect on our results. Second, although we strongly recommend participation in follow-up visits at every developmental stage and implement multi-faceted strategies to enhance follow-up rates across age groups (including health education, transportation cost reimbursement, and small incentives), dropout remains inevitable, potentially introducing selection bias. However, we performed a sensitivity analysis by restricting the study population to children with only one visit per age group. The consistent findings between this restricted cohort and the overall study population demonstrate the robustness of our results (Supplementary Table S5). Third, the proportion of girls in the NC group is higher than in the ART group. However, we conducted PSM matching based on the child’s age, gender, and parents’ age, as shown in Supplementary Tables S3 and S4. The results of PSM matching are similar to the conclusions of the overall population, which proves the robustness of our results. We believe that the predominance of female participants in the NC group is unlikely to have significantly influenced our study outcomes.

## Conclusion

The findings of this study indicate that children conceived via ART exhibit a thyroid function profile comparable to that of children conceived naturally. Furthermore, ART appears to be safe with respect to the long-term thyroid function of these children. Future studies should focus on tracking the thyroid function safety of children conceived via ART as they transition into adolescence and adulthood.

## Supplementary Material

hoag009_Supplementary_Data

## Data Availability

Data cannot be shared due to ethical approval constraints.
