# Supplementary material for: Lack of difference in thyroid hormone profile between offspring conceived naturally and through ART
Source: Hum Reprod Open. 2026 Feb 9;2026(1):hoag009. doi: 10.1093/hropen/hoag009 (PMC12949518; doi:10.1093/hropen/hoag009)
Supplement: hoag009_Supplementary_Data [file hoag009_supplementary_data.docx]

**
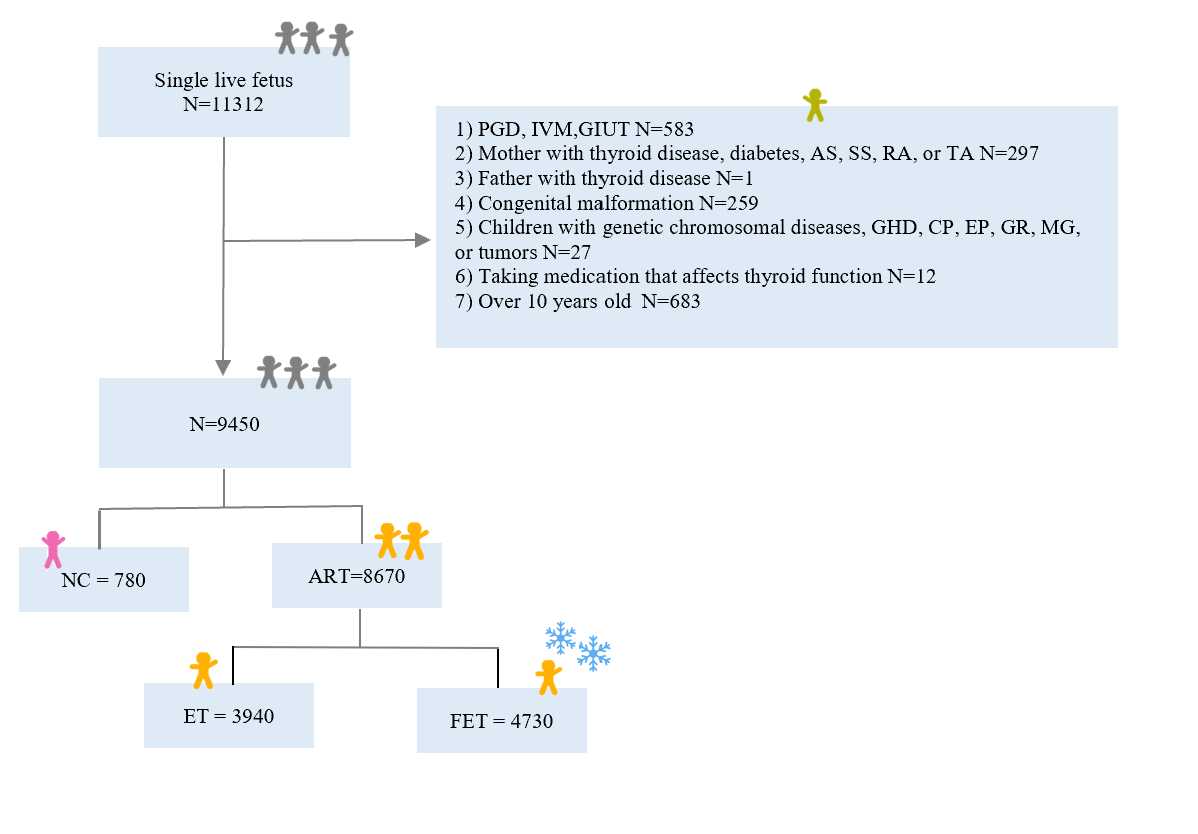
**

**Supplementary Figure S1: Flowchart**

Abbreviation: N, number; PGD, preimplantation genetic diagnosis; IVM, in vitro maturation; GIUT,

Gamete intrauterine transfer; AS, ankylosing spondylitis; SS, Sjogren's syndrome; RA, rheumatoid arthritis;

TA, takayasu arteritis; GHD, growth hormone deficiency dwarfism; CP, cerebral palsy; EP, epilepsy; GR,

growth retardation; MG, myasthenia gravis; NC, naturally conceived children; ART: children conceived

from assisted reproductive technology; ET, Fresh embryo transfer; FET, Frozen embryo transfer

**Supplementary Table S1:** **Follow-up Status at Each Follow-up Stage.**

|  | Number | Follow-up Visit | Follow-up Visit =1 | Follow-up Visit =2 | Follow-up Visit =3 |
| --- | --- | --- | --- | --- | --- |
| 1.5-2.9y | 4984 | 5372 | 4596 | 388 | 0 |
| 3-5.9y | 5183 | 6092 | 4295 | 867 | 21 |
| 6-10y | 1992 | 2397 | 1627 | 325 | 40 |

Abbreviation: y, year

**Supplementary Table S2: Follow-up Timing and Participant Count in Our Cohort**

| Follow-up timing | Number |
| --- | --- |
| *One of the follow-up stages: 1.5-2.9y, 3-5.9y or 6-10y | 6916 |
| 1.5-2.9y and 3-5.9y | 1515 |
| 3-5.9y and 6-10y | 736 |
| 1.5-2.9y, 3-5.9y and 6-10y | 175 |
| 1.5-2.9y and 6-10y | 108 |

*The child visits only during one of the follow-up stages: 1.5-2.9y, 3-5.9y

or 6-10y.

**Supplementary Table S3: Children Characteristics, Parental Characteristics, and Family Socioeconomic Status after PSM.**

|  | **NC**  **(570)** | **ET**  **(2321)** | **FET**  **(2410)** | **ET vs NC**  P-value | **FET vs NC**  P-value |
| --- | --- | --- | --- | --- | --- |
| Parental characteristics |  |  |  |  |  |
| Maternal age at delivery, y | 31.07±4.06 | 31.19±4.11 | 31.33±4.14 | 0.534 | 0.165 |
| Paternal age at delivery, y | 31.92±4.58 | 31.99±4.59 | 32.06±4.62 | 0.751 | 0.519 |
| GDM, n (%) | 19 (3.3%) | 155 (6.7%) | 192 (8.0%) | **0.001** | **<0.001** |
| HDP, n (%) | 11 (1.9%) | 87 (3.7%) | 147 (6.1%) | **0.013** | **<0.001** |
| Tobacco exposure during pregnancy | 7 (1.2%) | 65 (2.8%) | 64 (2.7%) | **<0.001** | **<0.001** |
| Parity, n (%) |  |  |  |  |  |
| First born | 226 (39.6%) | 1996 (86.0%) | 1940 (80.5%) | **<0.001** | **<0.001** |
| Second or later | 334 (58.6%) | 318 (13.7%) | 468 (19.4%) |  |  |
| Family socioeconomic status |  |  |  |  |  |
| Highest occupation |  |  |  |  |  |
| Mental labor | 218 (38.2%) | 712 (30.7%) | 760 (31.5%) | **<0.001** | **<0.001** |
| Physical labor | 342 (60.0%) | 1556 (67.0%) | 1584 (65.7%) |  |  |
| Student or unemployed | 7 (1.2%) | 52 (2.2%) | 66 (2.7%) |  |  |
| Highest education |  |  |  |  |  |
| College or above | 326 (57.2%) | 1022 (44.0%) | 1043 (43.3%) | **<0.001** | **<0.001** |
| Senior High school | 115 (20.2%) | 633 (27.3%) | 676 (28.0%) |  |  |
| Junior high school or below | 129 (22.6%) | 666 (28.7%) | 691 (28.7%) |  |  |
| Child characteristics |  |  |  |  |  |
| Male, n (%) | 275 (48.2%) | 1145 (49.3%) | 1213 (50.3%) | 0.676 | 0.396 |
| Birth weight, g | 3434.72±477.84 | 3429.17±500.37 | 3469.72±520.56 | 0.811 | 0.143 |
| Length, cm | 50.38±1.86 | 50.33±1.89 | 50.22±2.02 | 0.554 | 0.073 |
| Gestational age, w | 39.17 (1.41%) | 39.14 (1.44%) | 39.01 (1.61%) | 0.647 | 0.032 |
| Premature birth, n (%) | 0.04 (0.19%) | 0.05 (0.22%) | 0.07 (0.25%) | 0.130 | **0.009** |
| Age, y | 3.99±2.01 | 3.97±1.84% | 4.06±2.01 | 0.828 | 0.431 |
| 1.5-2.9y, n (%) | 291 (40.5%) | 1041 (36.2%) | 1125 (39.1%) | 0.088 | 0.176 |
| 3-5.9y, n (%) | 316 (44.0%) | 1381 (48.1%) | 1224 (42.5%) |  |  |
| 6-10y, n (%) | 111 (15.5%) | 450 (15.7%) | 531 (18.4%) |  |  |
| BMI, kg/m2 | 15.98±1.72 | 16.21±2.05 | 16.36±2.24 | **0.007** | **<0.001** |

Data presented as mean ± SD for continuous variables and n (%) for categorical variables;

Abbreviations: PSM, propensity score matching; NC, naturally conceived children; ET, Fresh embryo transfer; FET,

Frozen embryo transfer;

BMI: body mass index; GDM: gestational diabetes mellitus; HDP: hypertensive disorders during pregnancy;

Matched the children's age, sex and parental age at delivery.

Bolded variables indicate statistical significance (p≤0.05);

**Supplementary Table S4: Differences in Thyroid Function between NC and ART Children after PSM.**

|  | **NC** | **ET** | **FET** | **ET vs NC** | | **FET vs NC** | |
| --- | --- | --- | --- | --- | --- | --- | --- |
|  |  |  |  | Unadjusted  β (95% CI) | Adjusted  β (95% CI) | Unadjusted  β (95% CI) | Adjusted  β (95% CI) |
| 1.5-2.9y, n | 291 | 1041 | 1125 |  |  |  |  |
| TSH, uIU/mL | 2.72 ±1.28 | 2.94 ±1.42 | 2.77 ±1.33 | **0.24(0.05, 0.42)** | **0.27(0.07, 0.47)** | 0.07(-0.11, 0.24) | 0.10(-0.09, 0.29) |
| FT3, pmol/L | 6.97±0.86 | 6.98 ±0.81 | 6.99 ±0.81 | 0.01(-0.10, 0.13) | 0.02(-0.10, 0.14) | 0.02(-0.10, 0.13) | 0.02(-0.10,0.14) |
| FT4, pmol/L | 18.51±1.94 | 18.70±2.12 | 18.69 ±1.99 | 0.20(-0.07, 0.47) | 0.27 (-0.03,0.56) | 0.19 (-0.07, 0.45) | 0.26(-0.03, 0.54) |
| 3-5.9y, n | 316 | 1381 | 1224 |  |  |  |  |
| TSH, uIU/mL | 3.01±1.28 | 3.15 ±1.54 | 3.07 ±1.45 | 0.15 (-0.02,0.32) | 0.10 (-0.09, 0.29) | 0.07 (-0.10, 0.24) | 0.01 (-0.18,0.20) |
| FT3, pmol/L | 6.82 ±0.79 | 7.02 ±0.92 | 7.04±0.92 | **0.20 (0.10, 0.30)** | **0.18 (0.07,0.30)** | **0.22 (0.12, 0.33)** | **0.19 (0.08,0.31)** |
| FT4, pmol/L | 18.59 ±2.06 | 18.66 ±2.03 | 18.65±2.08 | 0.05(-0.22, 0.32) | -0.002 (-0.32, 0.31) | 0.05 (-0.22, 0.32) | 0.01(-0.30, 0.31) |
| 6-10y, n | 111 | 450 | 531 |  |  |  |  |
| TSH, uIU/mL | 3.26±1.52 | 3.29 ±1.49 | 3.26 ±1.54 | 0.02 (-0.31, 0.34) | -0.04 (-0.38, 0.30) | -0.01 (-0.35, 0.32) | -0.08 (-0.42, 0.27) |
| FT3, pmol/L | 6.75 ±0.70 | 6.85 ±0.84 | 6.85 ±0.82 | 0.12 (-0.04,0.28) | 0.10 (-0.05, 0.27) | 0.11(-0.05,0.28) | 0.09 (-0.07,0.26) |
| FT4, pmol/L | 18.55±2.34 | 18.32 ±1.99 | 18.39 ±2.08 | -0.21 (-0.71, 0.28) | -0.29 (-0.78, 0.21) | -0.13 (-0.63, 0.37) | -0.14 (-0.63,0.36) |

Abbreviations: n, number of follow-up visits; PSM, propensity score matching; NC, naturally conceived children; ET, Fresh embryo transfer; FET, Frozen embryo transfer;

from assisted reproductive technology; TSH: thyroid stimulating hormone; FT3: free triiodothyronine; FT4: free tetraiodothyronine;

Bolded variables indicate statistical significance (p≤0.05);

Adjusted for children's age and BMI; parity; parental age at delivery; socioeconomic factors.; tobacco exposure during pregnancy.

**Supplementary Table S5: Differences in Thyroid Function between NC and ART Children.**

|  | **NC** | **ET** | **FET** | **ET vs NC** | | **FET vs NC** | |
| --- | --- | --- | --- | --- | --- | --- | --- |
|  |  |  |  | Unadjusted  β 95% CI | Adjusted  β 95% CI | Unadjusted  β 95% CI | Adjusted  β 95% CI |
| 1.5-2.9y, n | 278 | 1811 | 2895 |  |  |  |  |
| TSH, uIU/mL | 2.72 ±1.29 | 2.92±1.41 | 2.80 ±1.33 | **0**.**20(0.03, 0.38)** | **0.19(0.01, 0.37)** | 0.08(-0.09,0.25) | 0.07 (-0.11, 0.24) |
| FT3, pmol/L | 6.96±0.87 | 6.99 ±0.88 | 7.01 ±0.81 | 0.03(-0.07, 0.14) | 0.04 (-0.07, 0.15) | 0.05(-0.05, 0.15) | 0.05(-0.05, 0.16) |
| FT4, pmol/L | 18.48 ±1.97 | 18.75±2.56 | 18.66 ±2.04 | 0.28 (-0.01, 0.56) | **0.31(0.01, 0.61)** | 0.19 (-0.09, 0.46) | 0.20 (-0.09, 0.49) |
| 3-5.9y, n | 287 | 2345 | 2551 |  |  |  |  |
| TSH, uIU/mL | 2.98±1.26 | 3.16 ±2.51 | 3.06 ±1.48 | 0.18 (-0.06, 0.43) | 0.20 (-0.06, 0.47) | 0.09(-0.16, 0.33) | 0.10 (-0.15, 0.36) |
| FT3, pmol/L | 6.82±0.78 | 6.99 ±0.88 | 7.04 ±0.90 | **0.17 (0.06, 0.28)** | **0.19 (0.08, 0.30)** | **0.22** **(0.11, 0.33)** | **0.23 (0.11, 0.34)** |
| FT4, pmol/L | 18.66 ±2.04 | 18.60 ±2.06 | 18.73 ±2.15 | -0.05(-0.31, 0.20) | -0.05 (-0.33, 0.22) | 0.07(-0.19, 0.33) | 0.08 (-0.19, 0.35) |
| 6-10y, n | 316 | 1028 | 648 |  |  |  |  |
| TSH, uIU/mL | 3.39 ±1.54 | 3.26±1.43 | 3.20 ±1.42 | -0.12 (-0.31, 0.06) | -0.17 (-0.39, 0.04) | -0.19 (-0.39, 0.002) | **-0.27 (-0.50, -0.04)** |
| FT3, pmol/L | 6.76 ±0.77 | 6.82 ±0.81 | 6.85 ±0.80 | 0.06 (-0.04, 0.16) | 0.05(-0.07, 0.16) | 0.09 (-0.02, 0.20) | 0.05(-0.07, 0.17) |
| FT4, pmol/L | 18.22 ±2.33 | 18.34 ±2.02 | 18.48 ±2.06 | 0.13 (-0.14, 0.39) | -0.14(-0.46, 0.17) | 0.27 (-0.02, 0.55) | -0.02(-0.35, 0.31) |

Included children who participate in only one follow-up visit in each age group.

Abbreviations: n, number of follow-up visits; NC, naturally conceived children; ET, Fresh embryo transfer; FET, Frozen embryo transfer; TSH: thyroid stimulating hormone; FT3:

free triiodothyronine; FT4: free tetraiodothyronine;

Bolded variables indicate statistical significance (p≤0.05);

Adjusted for children's age and BMI; parity; parental age at delivery; socioeconomic factors.; tobacco exposure during pregnancy.

**Supplementary Table S6: Differences in Thyroid Function between NC and ART Children.**

|  | **NC** | **ET** | **FET** | **ET vs NC** | | **FET vs NC** | |
| --- | --- | --- | --- | --- | --- | --- | --- |
|  |  |  |  | Unadjusted  β 95%CI | Adjusted  β 95%CI | Unadjusted  β 95%CI | Adjusted  β 95%CI |
| 1.5-2.9y, n | 293 | 1948 | 3131 |  |  |  |  |
| TSH, uIU/mL | 2.72±1.28 | 2.93±1.41 | 2.80±1.32 | **0.21(0.03, 0.40)** | **0.25(0.07, 0.42)** | 0.09(-0.06, 0.24) | 0.12(-0.04, 0.28) |
| FT3, pmol/L | 6.97±0.86 | 7.00±0.87 | 7.01±0.81 | 0.03(-0.08, 0.14) | 0.02 (-0.09, 0.14) | 0.05(-0.06, 0.16) | 0.04 (-0.07, 0.15) |
| FT4, pmol/L | 18.51±1.96 | 18.74±2.53 | 18.68±2.05 | 0.23(-0.02, 0.49) | 0.26 (-0.03, 0.54) | 0.17(-0.08, 0.41) | 0.17 (-0.09, 0.43) |
| 3-5.9y, n | 323 | 2773 | 2996 |  |  |  |  |
| TSH, uIU/mL | 3.01±1.28 | 3.17±2.38 | 3.09±1.47 | 0.17(0, 0.34) | 0.19 (-0.03, 0.41) | 0.10(-0.06, 0.25) | 0.10 (-0.07, 0.28) |
| FT3, pmol/L | 6.82±0.78 | 7.00±0.87 | 7.04±0.89 | **0.18(0.09,** **0.27)** | **0.19 (0.09, 0.29)** | **0.23(0.13, 0.32)** | **0.22(0.12, 0.32)** |
| FT4, pmol/L | 18.60±2.08 | 18.59±2.08 | 18.73±2.14 | 0 (-0.24, 0.24) | -0.03 (-0.30, 0.25) | 0.14(-0.10, 0.38) | 0.12 (-0.16, 0.39) |
| 6-10y, n | 239 | 918 | 547 |  |  |  |  |
| TSH, uIU/mL | 3.34 ±1.48 | 3.31±1.46 | 3.27 ±1.51 | -0.04(-0.26, 0.17) | 0.09(-0.15, 0.33) | -0.09(-0.32, 0.15) | 0.005(-0.25,0.26) |
| FT3, pmol/L | 6.72±0.77 | 6.81±0.81 | 6.87±0.82 | 0.08(-0.03,0.18) | 0.07(-0.06, 0.19) | **0.13(0.02, 0.25)** | 0.08(-0.05, 0.22) |
| FT4, pmol/L | 18.25±2.15 | 18.33±2.02 | 18.44 ±2.06 | 0.08(-0.22,0.37) | 0.02(-0.30, 0.35) | 0.18(-0.13, 0.50) | 0.10(-0.25, 0.45) |

Only included pre-adolescent children.

Abbreviations: n, number of follow-up visits; NC, naturally conceived children; ET, fresh embryo transfer; FET, frozen embryo transfer; TSH: thyroid stimulating hormone;

FT3: free triiodothyronine; FT4: free tetraiodothyronine;

Bolded variables indicate statistical significance (p≤0.05);

Adjusted for children's age and BMI; parity; parental age at delivery; socioeconomic factors.; tobacco exposure during pregnancy.

**Supplementary Table S7: Differences in Thyroid Function between ET and FET Children.**

|  | **ET** | **FET-NC** | **FET-HRT** | **ET vs FET-NC** | | **ET vs FET-** **HRT** | |
| --- | --- | --- | --- | --- | --- | --- | --- |
|  |  |  |  | Unadjusted  β 95%CI | Adjusted  β 95%CI | Unadjusted  β 95%CI | Adjusted  β 95%CI |
| 1.5-2.9y, n | 1948 | 1653 | 1122 |  |  |  |  |
| TSH, uIU/mL | 2.93±1.41 | 2.78 ±1.31 | 2.80±1.33 | **-0.14(-0.23, -0.05)** | **-0.14(-0.23, -0.05)** | **-0.14(-0.24, -0.04)** | **-0.14(-0.25, -0.04)** |
| FT3, pmol/L | 7.00±0.87 | 7.01±0.83 | 7.00±0.81 | 0.01(-0.04, 0.07) | 0.01(-0.04, 0.07) | -0.01(-0.06, 0.07) | -0.01 (-0.07, 0.06) |
| FT4, pmol/L | 18.74±2.53 | 18.69±2.11 | 18.69±2.11 | -0.06(-0.22, 0.09) | -0.06(-0.22, 0.10) | -0.09(-0.26, 0.07) | -0.11(-0.27, 0.06) |
| 3-5.9y, n | 2773 | 1545 | 1080 |  |  |  |  |
| TSH, uIU/mL | 3.17±2.38 | 3.07±1.44 | 3.08±1.50 | -0.12 (-0.24, 0.01) | -0.11(-0.23, 0.01) | -0.10 (-0.24, 0.03) | -0.12 (-0.25, 0.02) |
| FT3, pmol/L | 7.00±0.87 | 7.00±0.84 | 7.11±0.99 | -0.001(-0.06**,** 0.05) | -0.004(-0.06, 0.05) | **0.12(0.05, 0.19)** | **0.09 (0.02, 0.16)** |
| FT4, pmol/L | 18.59±2.08 | 18.62±2.06 | 18.85±2.26 | 0.03(-0.11, 0.16) | 0.02 (-0.11, 0.16) | **0.25 (0.09, 0.42)** | **0.26** **(0.09, 0.42)** |
| 6-10y, n | 1281 | 342 | 249 |  |  |  |  |
| TSH, uIU/mL | 3.28±1.45 | 3.28±1.52 | 3.22±1.49 | 0.03 (-0.16, 0.21) | 0 (-0.18, 0.18) | -0.15 (-0.36, 0.05) | **-0.21 (-0.41, -0.003)** |
| FT3, pmol/L | 6.79±0.79 | 6.89±0.80 | 6.87±0.78 | 0.09 (-0.01, 0.19) | 0.07 (-0.03, 0.17) | 0.07 (-0.04, 0.18) | 0.02 (-0.09,0.12) |
| FT4, pmol/L | 18.27±2.02 | 18.52±2.09 | 18.47±2.03 | **0.29 (0.03, 0.54)** | 0.22 (-0.04, 0.47) | 0.18 (-0.10, 0.47) | 0.16 (-0.12, 0.44) |

Data presented as mean ± SD for continuous variables and n for categorical variables;

Abbreviations: n, number of follow-up visits; ET, fresh embryo transfer; NC-FET: Natural cycle protocol for frozen embryo transfer; HRT-FET: Hormone replacement therapy for frozen

embryo transfer; TSH: thyroid stimulating hormone; FT3: free triiodothyronine; FT4: free tetraiodothyronine;

Bolded variables indicate statistical significance (p≤0.05);

Adjusted for children's age and BMI; parity; parental age at delivery; socioeconomic factors.; tobacco exposure during pregnancy.
